# Exploiting Pharma 4.0 Technologies in the Non-Biological Complex Drugs Manufacturing: Innovations and Implications

**DOI:** 10.3390/pharmaceutics15112545

**Published:** 2023-10-28

**Authors:** Vera Malheiro, Joana Duarte, Francisco Veiga, Filipa Mascarenhas-Melo

**Affiliations:** 1 Drug Development and Technology Laboratory, Faculty of Pharmacy, University of Coimbra, Pólo das Ciências da Saúde, Azinhaga de Santa Comba, 3000-548 Coimbra, Portugal; veracatarina.am13@gmail.com (V.M.); joanainesduarte@hotmail.com (J.D.); fveiga@ff.uc.pt (F.V.); 2 LAQV, REQUIMTE, Department of Pharmaceutical Technology, Faculty of Pharmacy, University of Coimbra, Pólo das Ciências da Saúde, Azinhaga de Santa Comba, 3000-548 Coimbra, Portugal; 3 Higher School of Health, Polytechnic Institute of Guarda, Rua da Cadeia, 6300-307 Guarda, Portugal

**Keywords:** Pharma 4.0, non-biological complex drugs, three-dimensional (3D) printing, digital twins, in silico modeling, machine learning

## Abstract

The pharmaceutical industry has entered an era of transformation with the emergence of Pharma 4.0, which leverages cutting-edge technologies in manufacturing processes. These hold tremendous potential for enhancing the overall efficiency, safety, and quality of non-biological complex drugs (NBCDs), a category of pharmaceutical products that pose unique challenges due to their intricate composition and complex manufacturing requirements. This review attempts to provide insight into the application of select Pharma 4.0 technologies, namely machine learning, in silico modeling, and 3D printing, in the manufacturing process of NBCDs. Specifically, it reviews the impact of these tools on NBCDs such as liposomes, polymeric micelles, glatiramer acetate, iron carbohydrate complexes, and nanocrystals. It also addresses regulatory challenges associated with the implementation of these technologies and presents potential future perspectives, highlighting the incorporation of digital twins in this field of research as it seems to be a very promising approach, namely for the optimization of NBCDs manufacturing processes.

## 1. Introduction

It is widely recognized that bringing a new pharmaceutical drug to the market is a complex, lengthy, and costly process associated with high uncertainty. This process is known as drug development and encompasses various stages, including preclinical research, drug design and production, regulatory filling, clinical trials in humans, obtaining regulatory approval, and the subsequent steps of manufacturing and marketing [1]. Throughout the years, the pharmaceutical industry has undergone significant changes and advancements, progressing from Pharma 1.0 to Pharma 4.0, and more recently reaching the era of Pharma 5.0. Even though Pharma 4.0 is a relatively recent development, the truth is that certain pharmaceuticals are already venturing into the 5.0 era [2].

During Pharma 1.0, the processing of materials derived from minerals, animals, and plants underwent a significant transformation. The use of basic hand-operated tools gave way to the introduction of commercial-scale equipment capable of crushing, blending, milling, and pressing a larger quantity of medicines. In fact, certain key machines developed during the Pharma 1.0 era continue to be widely used in the present day, showcasing their durability and effectiveness [3]. Subsequently, electricity and early electronic machines ushered in a new era in the evolution of the pharmaceutical industry. This phase witnessed the integration of digital tools into various aspects of pharmaceutical research, development, manufacturing, and distribution [4]. For the pharmaceutical sector, this technological incorporation marked a significant milestone because it allowed for a more data-driven and patient-centric approach, besides providing a larger-scale production and more efficient quality control. However, these process controls were far from perfect, since they were confined to pre-determined and static settings, which only allowed for the monitoring of process performance and passive control strategies [3]. The third industrial revolution was enabled by the emergence of computers and communication technologies, including networked computing, the internet, and wireless communications [4]. This development resulted in increased automation of both processes and equipment, fostering the implementation of continuous manufacturing and enhanced active control. This revolution, known as Pharma 3.0, enabled the adoption of advanced control strategies, elevating product and process quality and reducing the need for human operators. This decrease in reliance on human operators also streamlined the tracking of parameters and production metrics. Although some industries are well into Industry 3.0, the pharmaceutical industry is still very much transitioning into it [3]. Pharma 3.0 also brought the implementation of advanced process analytical technology (PAT), which provides real-time data on process and product quality. It also enhanced quality-by-design (QbD) processes, which focus on controlling product quality within specific parameters [3]. While Pharma 3.0 already enables a much-improved understanding of how to capture, analyze, and secure large amounts of data in pharmaceutical manufacturing, there is still room for further technological advancements to achieve the full potential of PAT and QbD [5]. Later on, with the appearance of artificial intelligence (AI), cloud computing, machine learning (ML), big data analytics, in silico modeling, 3D printing, and other advanced manufacturing technologies, the manufacturing process was actualized, forcing the emergence of another industrial revolution, known as Pharma 4.0 [6]. These advanced manufacturing technologies empower autonomous and self-organizing systems capable of operating independently without human intervention [3].

In the pharmaceutical sector, the fourth industrial revolution allowed for a shift in the paradigm of formulation development [7]. The potential to integrate diverse data sources facilitates the connection of both external and internal information, establishing a comprehensive network. In the context of pharmaceutical manufacturing, this integration entails merging external data, including patient experiences, market demands, supplier inventories, and public health emergencies, with internal data encompassing energy and resource management, modeling and simulation results, and laboratory data. This fusion enables unparalleled real-time responsiveness, monitoring, control, and predictive capabilities [3].

In an ever-evolving marketplace characterized by a fast-changing and smartly integrated supply chain, coupled with more active participation of patients in their healthcare decision-making, pharmaceutical companies face an urgent imperative to maintain their competitive advantage. In this setting, the key factor that sets businesses apart is their ability to meet the expectation of Pharma 4.0, which, despite being a complex process to implement, provides enhanced resources for ensuring product safety compliance, safeguarding the supply chain, and fostering pharmaceutical development [6]. This concept of Pharma 4.0 is closely linked to artificial intelligence (AI), a technology-based system involving various advanced tools and networks that can mimic human intelligence [3]. It possesses systems and software with the ability to interpret and learn from the input data, enabling them to autonomously make decisions in order to achieve specific objectives [8]. AI plays a crucial role in Pharma 4.0 by enabling smart, data-driven decision-making and optimization throughout the entire pharmaceutical value chain [9].

Non-biological complex drugs (NBCDs) are complex, often comprising intricate structures and diverse components [10]. Therefore, traditional quality control methods may not fully capture the complexities of these drugs. Pharma 4.0 techniques can offer real-time monitoring and predictive quality control, allowing for more effective detection of deviations and potential issues. Besides, since NBCD manufacturing involves handling a vast amount of data from multiple stages of the production process, these innovative technologies can leverage this data to support data-driven decision-making, enabling manufacturers to identify patterns, trends, and correlations that may impact product quality, stability, and reproducibility [11]. In this sense, this manuscript presents a view on the application of select Pharma 4.0 technologies, namely machine learning, in silico modeling, and 3D printing, for enhancing the overall efficiency, safety, and quality of non-biological complex drugs (NBCDs), a category of pharmaceutical products that pose unique challenges due to their intricate composition and complex manufacturing requirements.

However, it is important to keep in mind that to attain Pharma 4.0, while embracing cutting-edge manufacturing technologies, it is also important to simultaneously overcome regulatory obstacles [12]. In this context, this review not only addresses these regulatory challenges but also explores potential future perspectives, notably the incorporation of Digital Twins in the development and manufacturing of non-biological complex drugs (NBCDs).

## 2. Emerging Tools of Pharma 4.0

**Figure 1:**
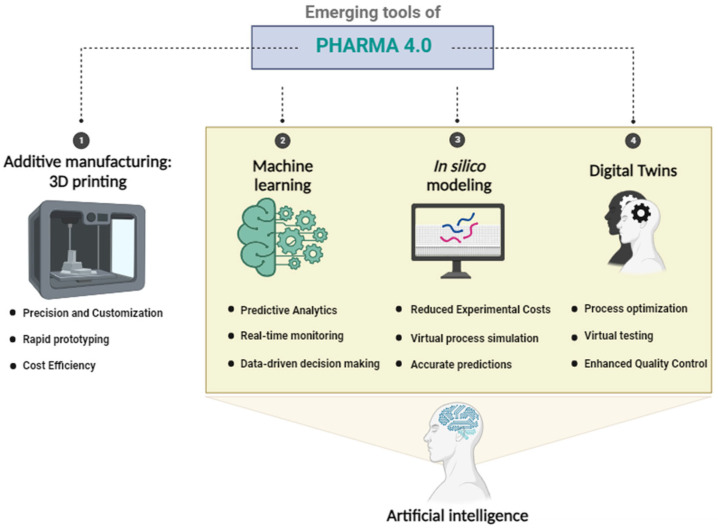
Emerging tools of Pharma 4.0 and their main advantages in drug manufacturing.

The following sections of this review will highlight and describe emerging tools of Pharma 4.0, such as additive manufacturing (3D printing), in silico modeling, and machine learning, which are represented in Figure 1.

### 2.1. Additive Manufacturing: Three-Dimensional (3D) Printing

Additive manufacturing encompasses all manufacturing techniques that involve the sequential addition of material to build a 3D object. Typically, a digital representation of the desired object is created using computer-aided design software; subsequently, the product is produced using one or more additive technologies [13].

3D printing, as an additive manufacturing technique, holds immense potential within the pharmaceutical sector. It consists of the layer-by-layer creation of 3D objects based on digital designs and holds promise for the development of versatile release models. These models aim to address clinical needs and facilitate patient-centric treatment, including personalized dosing tailored to specific disease states or patient populations [14]. The classification of 3D printing techniques can be simplified into three main categories: printing-based inkjet systems, nozzle-based deposition systems, and laser-based writing systems [15].

Spritam^®^ (levetiracetam), an epilepsy medication developed by Aprecia Pharmaceuticals, was the first 3D-printed pharmaceutical to receive approval from the Food and Drug Administration (FDA) [16]. It utilizes a unique 3D printing technology called ZipDose^®^, which allows for the production of a highly porous tablet that dissolves rapidly in a small amount of liquid. This orodispersible tablet, characterized by its porous and soluble matrix composition, demonstrated similar pharmacological efficacy comparable to conventional tablets. On the other hand, this tablet exhibited a significant solubilization time improvement, indicative of faster dissolution [15]. It is worth noting that the approval of Spritam^®^ as a 3D-printed pharmaceutical by the FDA was a significant milestone in the field of pharmaceutical manufacturing, as it demonstrated the potential of 3D printing technology in the production of unique drug formulations and opened up possibilities for personalized medicine [16].

Extensive research has been dedicated to advancing the use of 3D printing techniques in pharmaceutical manufacturing. As these endeavors progress, additive manufacturing technology has not only demonstrated its potential for creating personalized medications tailored to individual patients but has also emerged as a viable option for large-scale manufacturing, thanks to its ability to generate dosage forms with unique performance characteristics, which would be difficult to achieve via traditional methods [17].

#### Advancements in the Pharmaceutical Industry Offered by 3D Printing Technology

In the pharmaceutical industry, 3D printing offers the benefit of creating products with sophisticated external shapes. In fact, this technique was employed to produce orally disintegrating printlets specifically tailored for individuals with visual impairment. These printlets were meticulously designed with Braille and Moon patterns on their surfaces, allowing patients to identify medications even when removed from their original packaging. This approach shows the potential to enhance accessibility and independence for visually impaired patients in managing their medication regimens, thereby reducing potential errors [18].

3D printing has also demonstrated unprecedented potential in controlling drug release and influencing drugs’ pharmacokinetic profiles. In fact, the use of 3D printing techniques was leveraged to produce multiparticulates, known as miniprintlets, loaded with paracetamol, which exhibited prolonged drug release patterns, offering a novel approach to drug delivery with remarkable flexibility and control over release properties. By adjusting parameters such as dimensions and matrix composition, the therapeutic effect can be finely tuned, enabling the production of multi-drug systems. Furthermore, the possibility of creating miniprintlets containing two drugs, namely paracetamol and ibuprofen, was also investigated. A miniprintlet utilizing different polymers was created, so that one drug was released immediately and the second was sustained over an extended period [19].

Another promising advantage of 3D-printing is the ability to delay drug release, reaching even the colon in the gastrointestinal (GI) tract. This advancement holds tremendous potential as a breakthrough for the treatment of inflammatory bowel diseases [17,20].

For its numerous advantages, this technology has been employed to manufacture an extensive range of pharmaceutical products. These includes printlets (3D printed tablets), capsules, subcutaneous implants, and transdermal microneedles, among other innovative drug delivery devices [14,21,22].

### 2.2. In Silico Modeling

In silico modeling refers to the use of computational methods and computer simulations to study and analyze complex systems, typically in the fields of science, engineering, and medicine [23].

This approach involves creating virtual representations or models of real-world systems, based on mathematical algorithms, statistical techniques, and biological or physical principles. By simulating the behavior of these models, researchers can gain insights into underlying mechanisms, predict outcomes, and test hypotheses without the need for costly or time-consuming physical experiments [24].

In the pharmaceutical sector, in silico modeling is used to predict the behavior of drug molecules, their interactions with biological targets, and their potential efficacy and safety profiles [25]. From this context arises the term “in silico trials”, which refers to the application of computer modeling and simulation to assess the safety and effectiveness of medicinal products, including drugs, medical devices, diagnostic products, and advanced therapy medicinal products [23].

Over the years, numerous reviews have described how in silico methods can be used for predicting the absorption, distribution, metabolism, excretion, and toxicity (ADME/TOX) of different drugs [26]. This plays a crucial role in assisting decision-making, since it is not possible to experimentally test all possible combinations of drug interactions [27]. By simulating the behavior of substances in silico, researchers can quickly identify compounds with unfavorable ADME/TOX profiles and prioritize those with higher chances of success for further experimental evaluation, thus saving time and resources and reducing the risk of costly failures in later stages of drug development [26].

This technique also provides a mechanistic insight into the underlying processes and interactions involved in ADME/TOX because it can simulate the behavior of substances at the molecular level, allowing researchers to understand the interactions between transporters, enzymes, receptors, and other factors influencing ADME/TOX properties. Such information can help in understanding the mechanisms of action, potential toxicities, and the design of safer and more effective drugs [28].

In addition, in silico models can also be employed to study complex biological systems, such as cellular processes and signaling pathways. These models can help unravel the underlying mechanisms governing these systems and aid in the understanding of diseases, as well as the development of therapeutic interventions [29,30].

As an illustrative example of the applicability of in silico modeling in the pharmaceutical field, one can consider the design of these models to predict and comprehend the mechanism of action of the human ether-a-go-go-related gene (hERG) blockers. This is relevant because the hERG gene encodes a crucial ion channel in the heart, namely he hERG potassium channel, which plays a crucial role in regulating the electrical activity of this organ. Certain drugs may inadvertently block the hERG channel, leading to a drug-induced QT prolongation, which can result in a potentially life-threatening heart rhythm disturbance called Torsade de Pointes. Therefore, understanding and predicting the effects of potential hERG blockers through in silico methods are vital in drug development and safety assessment [31].

### 2.3. Machine Learning (ML)

ML is an artificial intelligence-based technology that focuses on constructing computational models by training them with a set of data. It enables machines to automatically analyze and interpret complex patterns and data, improving their performance in making predictions or decisions. For instance, ML might predict the stability of a specific drug formulation by considering data from a range of previous experiments that investigated formulation stability [32].

Machine learning algorithms identify patterns, anomalies, and potential risks, enabling proactive quality management and enhancing accuracy, efficiency, and compliance, while ensuring high product standards, regulatory compliance, and patient safety [9]. In fact, by automating inspection processes, analyzing data for defects and deviations, and predicting quality outcomes, this AI technology is revolutionizing quality control and assurance processes.

This technology can be broadly categorized into three types: supervised learning, unsupervised learning, and reinforcement learning [33]. In the first one, the algorithm is supplied with training data that includes both recorded observations and their corresponding labels. With this information, the algorithm constructs a model that can predict the appropriate output label for new observations [34]. Unsupervised learning, on the other hand, deals with unlabeled data, where the algorithm aims to discover hidden patterns or structures in the data without any predefined labels. Basically, the model learns to cluster similar data points or find relationships between the variables [35]. Finally, reinforcement learning involves a learning approach that relies on the use of rewards to categorize inputs as “good” or “bad” data, based on their interaction with the surrounding environment. Through this process, the algorithm learns to optimize its actions to maximize positive outcomes and minimize negative ones [36].

The initial applications of machine learning in drug formulation date back to the 1990s when neural networks (NNs) were employed to forecast properties related to immediate-release oral tablets. This involved the preparation and assessment of a diverse array of tablet formulations and the training of the neural network with the acquired data, enabling the prediction of different outcomes such as disintegration time, dissolution rate, and friability. These early experiments marked the pioneering use of machine learning techniques in optimizing drug formulation processes [32].

In fact, regarding the machine learning domain, these neural networks remain the most used technique. However, when dealing with a small amount of data, this can be prone to overfitting due to the high complexity and risk of learning noise in the limited dataset. In such cases, alternative machine learning methods such as support vector machines (SVMs) and random forests can be more suitable choices.

SVMs represent a category of learning machines rooted in statistical learning theory. The fundamental concept behind employing SVMs for pattern classification can be summarized as follows. Initially, input vectors are mapped into a feature space, which can be of higher dimension, achieved via either linear or non-linear transformations. Subsequently, within this feature space, the objective is to identify an optimal linear division. This involves constructing a hyper-plane capable of effectively separating two classes with minimal error and maximal margin. The SVMs’ training process consistently strives for a globally optimized solution while preventing overfitting, rendering them proficient in managing a large number of features [37].

The random forest algorithm is a well-established machine learning technique which operates by creating decision trees using random subsets of the provided dataset. These trees are divided into nodes, which serve as decision points where the algorithm categorizes the given sample. As the data are processed, each decision tree generated by the algorithm makes an individual prediction, which is then compared against those of other trees. The final prediction is then determined based on the majority vote of the individual decision trees. This approach is particularly useful in handling small datasets effectively [38].

Nowadays, the application of ML in the healthcare sector allows for improved cancer diagnosis, the discovery of new antifibrotic and antibiotic molecules, and the development of self-driving laboratories. It also allows for the prediction of the products of certain chemical reactions and, consequently, the optimization of these reactions [32].

### 2.4. Digital Twins

In the pharmaceutical industry, digital twins can have several uses and take different forms and as such are being increasingly adopted to improve efficiency, productivity, and quality control of drug manufacturing processes. For instance, researchers have developed computer simulations to create digital twins of dissolution apparatuses, namely USP II, and tablets to mimic their behavior realistically. By using these digital twins, they could investigate drug release profiles and shear rates that act on the tablet, under different paddle speeds, in both USP II and biorelevant colon models. The objective was to understand how the USP II could be operated to achieve more realistic hydrodynamic conditions that resemble the conditions in the human colon in vivo. To ensure accuracy and relevance in the simulations, the behavior of the tablet and the motility patterns within the colon are derived from experimental data and in vivo observations, respectively. Based on their findings, the researchers recommend using an “on–off” operating mode in the USP II rather than a constant paddle speed. This “on–off” mode generates shear rate peaks, which better reflect the in vivo conditions of the human colon. This change in operating mode can help achieve more realistic simulation results, providing deeper insights into the tablet’s disintegration and drug release processes in colon-targeted solid dosage forms [39].

These digital twins were also employed as a digital representation of the continuous manufacturing process for pharmaceuticals. This allows for the prediction of critical process parameters (such as those in the feeders, blender, and tablet press) and critical quality attributes (tablet composition, weight, thickness, and hardness) throughout the manufacturing process. This means that pharmaceutical companies can study the steady-state operation of the process within the design space; examine the impact of different operating conditions, materials, and process parameters; and understand dynamic response to disturbances or variations in the process [40].

In addition, researchers have developed a new and innovative concept called a “neural network–intelligent digital twin” to describe, predict, and optimize the outcomes of the manufacturing process of solidified nanosuspensions. The authors integrated the artificial neural network into the digital twin development process in a stepwise manner. The steps included data sampling, model deployment, and curve fitting. This means that the NN was trained using a dataset containing information about the manufacturing process, where the network´s architecture and parameters were optimized (curve fitting) to effectively represent the behavior of the process. One of the primary roles of the ANN within the digital twin is to augment the amount of available data. Since training a neural network typically requires a significant amount of data, the ANN generates additional simulated data points to further calibrate and validate the model. In the process, the digital twin can be fine-tuned to better represent the real-world manufacturing process, without the need for extensive physical experiments, while reducing uncertainties in the model´s predictions. Overall, this research introduces a sophisticated approach that combines NN technology and digital twin concepts to improve the understanding, prediction, and optimization of the manufacturing process for solidified nanosuspensions. This has the potential to lead to more efficient and cost-effective manufacturing processes in the pharmaceutical sector [41].

Another—and very recent—example of the applications of digital twins is the use of this technology, enabled by process analytical technology (PAT) approaches, to improve various aspects of the SARS–COVID-19 vaccine manufacturing process, such as capacity multiplication, reduction of out-of-specification batch failures, personnel training, efficient operation, optimal utilization of resources, and faster product release. Specifically, the focus is on messenger ribonucleic acid (mRNA) vaccine processing and the potential use of digital twins to address bottlenecks and optimize the process. Researchers have suggested creating a digital twin of the entire process which would convert plasmid deoxyribonucleic acid (pDNA) into mRNA. By combining digital twins with PAT, the vaccine production process can be enhanced, rendering it more efficient, and manufacturing capacity can be multiplied without compromising production quality and efficiency. In addition, researchers also believe that digital twins can also be used as a training tool for personnel to become qualified in operating the manufacturing process effectively and efficiently. The technology additionally facilitates the optimization of the utilization of scarce buffers and chemicals. This in turn leads to better resource management in the manufacturing process and a faster product release, enabling vaccines to be swiftly distributed to meet global demand [42].

In general, digital twins allow for enhanced drug formulation development, reduced reliance on costly experiments, and improved drug performance assessment. They enable pharmaceutical companies to move towards more data-driven and agile manufacturing processes, leading to better products and reduced costs. However, despite their potential benefits, the pharmaceutical industry has not fully embraced the use of digital twins in their operations [41].

## 3. Non-Biological Complex Drugs (NBCDs)

NBCDs are defined as medicinal products that are not derived from living organisms but are entirely synthesized via a fully synthetic process. They are also characterized by an active substance comprising multiple structures that cannot be entirely isolated, quantified, characterized, or fully described using physicochemical analytical methods [43].

**Figure 2:**
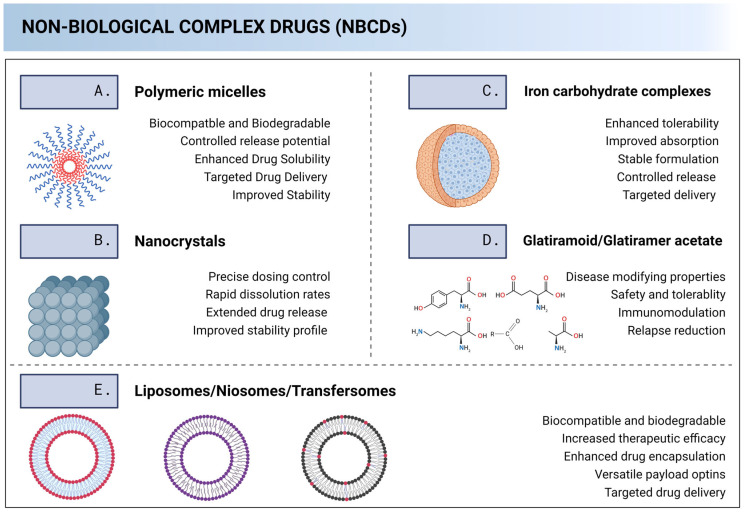
Illustrative depiction of non-biological complex drugs (NBCDs) and descriptions of their main technological advantages.

NBCDs are closely related to nanoparticles, and some examples include fat emulsions, liposomes, polymeric micelles, iron-carbohydrate complexes, dendrimers, swelling cross-linked polymers, and glatiramoids, among others. Sometimes, low molecular weight heparins, dry powder inhalers, ocular/intravenous emulsions, and dermal patches are seen as NBCDs as well [11]. Figure 2 illustrates the NBCDs that are within the scope of this manuscript.

In a conference that took place in Budapest (October 2014), Dr. De Vlieger listed a few key attributes for an NBCD [1]: (1) it consists of a multitude of closely related structures; (2) the entire complex is the active pharmaceutical ingredient; (3) the properties cannot be fully characterized via physicochemical analysis; and (4) a robust and well-controlled manufacturing process is key to reproducing the product.

Regarding NBCDs, one of their major challenges lies in the characterization process, specifically in evaluating batch-to-batch similarity and assessing the impact of manufacturing process variations. This sensitivity to manufacturing changes can give rise to challenges in ensuring reproducibility when produced by different manufactures. A noteworthy example of this occurred with follow-on products for iron sucrose complexes, where reports indicated that patient safety was compromised by switching patients to a follow-on product [44]. The complex nature of NBCDs may arise from various factors. For instance, the complexity may stem from the active substance itself, as seen with glatiramer acetate, or it may arise from the formulation, as observed with liposomes [10].

All of above explain why NBCDs fall under a distinct regulatory framework compared to small molecule drugs [45].

Much like biologics, the quality of NBCD products is intricately linked to a robust and meticulously controlled manufacturing process. This poses challenges for generic drug developers aiming to replicate NBCD products once the market exclusivity of the originator product has expired. Unlike biologics, which benefit from a well-established biosimilar pathway, NBCDs lack recognition as a distinct category of medicines. Consequently, a formal regulatory pathway for their approval has yet to be defined. Presently, a “case-by-case” approach is employed to regulate NBCD follow-on products in the EU. Additionally, NBCDs may undergo a non-centralized authorization procedure, decentralizing regulatory approval to competent national authorities. However, this decentralized approach can introduce heterogeneity in the regulatory assessment of NBCD follow-on products across the EU, potentially leading to varied outcomes and, in some instances, raising concerns related to safety and efficacy. For that reason, given the regulatory uncertainty still surrounding these products, we can only infer that by incorporating Pharma 4.0 tools, a more standardized and data-driven approach can be applied to the development and manufacturing of NBCDs. For example, the implementation of real-time monitoring systems, guided by Pharma 4.0 principles, enhances our understanding of critical manufacturing parameters for complex NBCDs, ensuring consistency in product attributes essential for establishing pharmaceutical equivalence. Data analytics tools analyze vast datasets, enabling trend identification and maintenance of product attributes. In silico modeling capabilities of Pharma 4.0 optimize NBCD formulation design, predicting and assessing variations to ensure bioequivalence. Machine learning algorithms enable adaptive control strategies during NBCD manufacturing, dynamically responding to variations and contributing to bioequivalence maintenance, particularly in the absence of a consistent regulatory framework. Aligning with the QbD principles of Pharma 4.0, a systematic QbD approach addresses factors affecting bioequivalence, providing a structured framework for regulatory assessments. In totality, Pharma 4.0 tools offer comprehensive solutions to bridge the current regulatory gap associated with NBCDs, promoting consistency and efficacy in their evaluation and approval processes. Moreover, the conventional generic approach, centered on establishing pharmaceutical equivalence and bioequivalence, has been instrumental in introducing numerous safe and effective alternatives to innovative medicines. Nevertheless, this approach has primarily been employed for products that can be thoroughly characterized. In the case of more intricate molecules characterized by inherent complexities and challenges in characterization, such NBCDs demonstrating bioequivalence may benefit from the exploration of alternative methodologies [46,47]. However, given the criticality of this issue, more research, more evidence and discussion are needed around it.

### 3.1. Polymeric Micelles

Polymeric micelles are nanoscale particles (sizes ranging between 20 and 200 nm) that consist of a hydrophobic core, serving as a reservoir for poorly soluble active pharmaceutical ingredients (APIs), and a hydrophilic shell that offers colloidal stability and limits protein adsorption and opsonization, leading to extended circulation times [48]. Since the physicochemical properties and the in vivo distribution, safety, and efficacy of the final product are highly dependent on the chosen polymer chemistry and manufacturing process, this explains why polymeric micelles are classified as NBCDs [49]. Despite the challenges associated with them, these structures hold great promise in drug manufacturing and delivery, as they offer several advantages which make them attractive for various therapeutic applications [48]. By encapsulating hydrophobic drugs in their core, these structures provide increased solubility and stability, facilitating their delivery to target sites. [49]. Simultaneously, these nanocarriers also have shown improved therapeutic outcomes when compared to the free drug [50]. In addition, the biocompatibility of many polymers used in micelle formulation makes them well tolerated by the body, reducing the risk of adverse reactions [48]. Moreover, by modifying the surface properties of the micelles, these can be engineered to selectively accumulate at specific disease sites, taking advantage of the enhanced permeability and retention effect of tumor tissues or inflamed regions [51].

More recently, researchers have been studying combinations of two or more polymers in order to assemble polymeric mixed micelles [52]. The underlying concept is to combine polymers with complementary characteristics to enhance drug encapsulation, stability, and targeted delivery. Overall, this is a direct and convenient approach to improve the physical stability and enhance the drug-loading capacities of conventional polymeric micelles for drug delivery [53].

### 3.2. Liposomes

Liposomes are colloidal and spherical vesicles composed of lipid molecules, primarily phospholipids, which have a hydrophilic head and hydrophobic tails, allowing them to form, in an aqueous environment, a closed bilayered structure [54]. They are classified according to their size, which can go from 0.025 µm (very small liposomes) to several µm (large liposomes), and their number of double layers [55]. The formulation of these structures involves the dispersion of the lipid molecules in an aqueous phase to form the vesicles. This can be carried out via mechanical methods (microfluidization, extrusion), replacement of organic solvents via aqueous media methods (ethanol injection, reverse phase evaporation), and detergent removal methods [54]. These structures have been studied in several pharmaceutical studies as drug delivery systems due to their remarkable capacity to encapsulate molecules with varying solubilities, namely that hydrophilic molecules can be accommodated in the internal core, hydrophobic molecules within the lipid bilayer, and amphiphilic molecules at the water/lipid bilayer interface. Moreover, their exceptional biocompatibility, biodegradability, lack of toxicity, and non-immunogenic nature further enhance their potential as an excellent therapeutic options [56].

Liposomes offer a host of additional benefits, including the capability to carry substantial drug loads, the capacity for self-assembly, and the versatility to modify a wide range of physicochemical and biophysical properties, thereby allowing for precise control over their biological characteristics [54]. It is therefore clear that liposomes are incredibly versatile structures that find applications across diverse fields. In fact, they can be found as a therapeutic option for cancer [57] as well for the treatment of skin conditions such as hyperpigmentation, among many others [58].

Over the years, diverse delivery systems based on liposomes have been developed, such as niosomes, transfersomes, ethosomes, and dendrossomes, of which we highlight the first two.

#### 3.2.1. Niosomes

Niosomes were initially developed for the cosmetic industry but have now a wide range of applications in the pharmaceutical field [59]. They consist of biodegradable and biocompatible vesicles composed of nonionic surfactants and, sometimes, cholesterol or its derivatives [60]. These structures allow us to overcome some limitations associated with liposomes, since they are not only more cost-effective and easier to prepare but also demonstrate a higher encapsulation efficiency [61]. This explains why niosomes have garnered significant attention in numerous studies for diverse applications, including for several types of cancers and fungal infections [62].

#### 3.2.2. Transfersomes

Transfersomes are specialized lipid-based nanocarriers composed of phospholipids and edge-active agents. These systems possess exceptional deformability and flexibility, allowing them to squeeze through the narrow pores of the stratum corneum [59]. This characteristic enables efficient transdermal drug delivery, offering a non-invasive alternative for systemic drug administration [63]. Transfersomes have garnered significant attention due to their potential to enhance drug absorption, minimize side effects, and improve patient compliance [64,65].

### 3.3. Glatiramoid/Glatiramer Acetate (GA)

Glatiramer acetate (GA), also known as Copolymer-1 or Cop-1, is a heterogeneous mixture of synthetic polypeptides, comprising L-alanine, L-lysine, L-glutamic acid, and L-tyrosine [66]. Its discovery took place in the late 1960s, when researchers were investigating the immunological properties of synthetic amino acid polymers in the quest for a synthetic antigen capable of inducing experimental autoimmune encephalomyelitis (EAE), the most commonly used model for studying multiple sclerosis (MS) [67]. In the US and Europe, GA has been approved as a disease-modifying treatment (DMT) for patients with relapsing forms of MS, a chronic inflammatory disease of the central nervous system (CNS) that starts as an autoimmune reaction leading to acute CNS inflammation, along with the disruption of myelin, ensheathing axons, and axonal damage [67].

Despite not being completely random, the amino acid sequences are not completely conserved from batch to batch, even when the process is tightly controlled. Therefore, the quality of these substances heavily relies on the precision of the manufacturing process [66].

GA falls under the category of NBCDs not only due to its intricate structure and variable composition, but also because fully characterizing and analyzing its molecular structure is exceptionally challenging compared to traditional small-molecule drugs [66,68].

### 3.4. Iron Carbohydrate Complexes Drugs

Iron carbohydrate complexes drugs are effective iron replacement agents for iron deficiency anemia treatment [69]. This is an area of great interest because it is the most common micronutrient deficiency worldwide [70].

Oral iron supplements in the form of ferrous iron may not be the perfect therapeutic approach due to their association with gastrointestinal intolerance, prolonged iron store repletion time, and impaired iron absorption. As a way to overcome these problems, intravenous iron–carbohydrate complexes appear to be an alternative approach that can lead to higher hemoglobin levels as well as faster replenishment of the body’s iron stores [71].

These structures consist of colloidal formulations composed of an iron core and a complex carbohydrate coating with an average particle size at the nanoscale. Different formulations differ in terms of the iron core size, carbohydrate shell coating material, and hydrodynamic size of the product. The stability of iron–carbohydrate complexes and the rate at which iron is released from the matrices vary among different products [72]. Furthermore, it is crucial to have a comprehensive knowledge of the physicochemical characteristics of the various intravenous iron–carbohydrate complexes to improve the safety and efficacy of the currently available products as well as for the formulation of new iron preparations in the future [69].

### 3.5. Nanocrystals

Nanocrystals are carrier-free drug particles with sizes in the nanometer range and crystalline characteristics [73]. Thanks to high drug loading, nanocrystals guarantee efficient drug delivery to cells or tissues and maintain potent therapeutic concentrations to achieve the desired pharmacological effect. Consequently, these transporters have become very attractive treatment options for various types of diseases [74].

This technology of nanocrystallization offers a very promising and effective way to deal with the low solubility and poor bio-availability of poorly soluble drugs because, due to their nanosize, they allow for increased dissolution pressure and a higher dissolution rate [73]. To produce nanocrystals, several methods have been implemented and they can be divided into three main categories: top-down (nanonization), bottom-up (crystal growth or nucleation), and combination techniques [75].

Capitalizing on the aforementioned advantages, these NBCDs have found application across various disease categories, such as in cancer therapy, inflammatory diseases (due to their capacity to improve on the physicochemical properties of Biopharmaceutics Classification System (BCS) class II drugs), and in the prevention of preterm birth (as progesterone is a BCS class IV drug, with poor solubility and very low permeability, which renders therapeutics difficult), among many other purposes [74,76].

## 4. Applying Pharma 4.0 Tools in the Production of Non-Biological Complex Drugs

### 4.1. Additive Manufacturing: 3D Printing

#### 4.1.1. Polymeric Micelles

**Figure 3:**
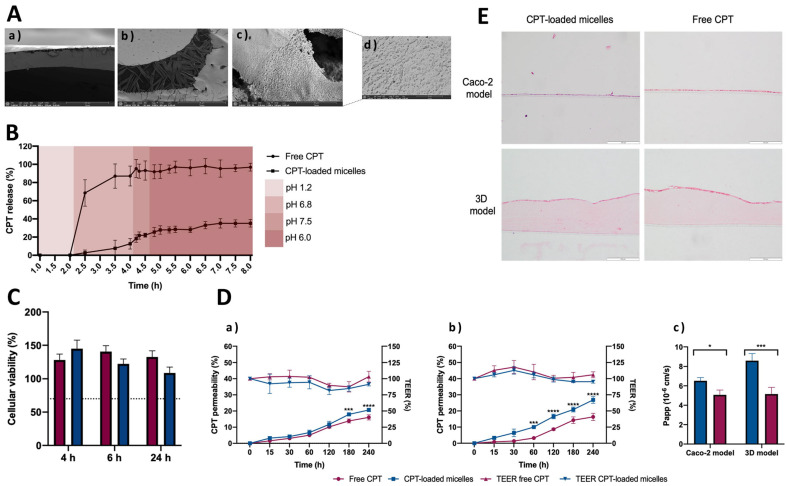
(**A**) Images obtained via scanning electron microscopy of (**a**) top layer of Eudragit in the printfills, (**b**) CPT crystals within the printfills, (**c**) micelles loaded with CPT inside of the printfills, and (**d**) magnified polymeric micelles inside the printfills. (**B**) Cumulative release of CPT from the printfills in simulated gastrointestinal fluids. The release of CPT-loaded micelles is depicted with squares, while the release of free CPT is indicated with circles. (**C**) The cell viability of the dissolution medium sourced from the printfills containing CPT-loaded micelles (depicted as blue columns) and free CPT (illustrated with pink columns) was assessed following 4, 6, and 24 h of incubation with Caco-2 cells. (**D**) Intestinal permeability and respective TEER values of CPT across a standard Caco-2 cell model (**a**) and across a 3D intestinal model (**b**) and apparent permeability coefficients of CPT-loaded micelles (blue) and free CPT (pink) in both Caco-2 monoculture model and the 3D model (**c**), where * *p* < 0.05, *** *p* < 0.001 and **** *p* < 0.0001 (**E**) H&AND staining to assess cellular integrity following exposure to the dissolution medium containing CPT-loaded micelles and free CPT during permeability assay. The cytoplasm was stained pink, while the nucleus was stained purple. The transwell membrane, positioned just beneath the cellular monolayer, remains transparent [77]. **CPT**: camptothecin; **H&AND**: hematoxylin, and eosin; **TEER**: transepithelial electrical resistance.

3D printing has exhibited promising results in the manufacturing of diverse drug delivery systems, such as polymeric micelles. In a recent investigation, chitosan-based polymeric micelles containing camptothecin (CPT) were integrated into 3D printing systems and coated with an enteric layer. This approach aimed to safeguard the nanosystems from the harsh conditions of the gastrointestinal tract [77]. The manufacturing process involved the use of a bioprinter that combines fused deposition modeling (FDM)—a widely used 3D printing method that involves melting and extruding filament materials layer by layer—and injection volume filling (IVF)—a technique that enables the incorporation of solutions or dispersions at room temperature into the extruded scaffold [77,78]. The internal structure of the printfills is depicted in Figure 3A. The in vitro drug release profile showed that both printfills containing the free drug and those with micelles effectively managed drug release. Consequently, there was no drug release from the printfills within the initial 2-h period at pH 1.2. However, when pH changed to 6.8, the retarding polymer began to dissolve, permitting the entry of water (Figure 3B). Researchers also concluded that both polymeric micelles and the free drug present in the dissolution media did not exhibit any cytotoxic effects on Caco-2 cells, a colorectal cancer cell line. In fact, these cells showed an increase in metabolic activity of up to 100%, which could be attributed to the presence of simulated gastrointestinal fluids (Figure 3C). Furthermore, as illustrated in Figure 3D, the permeability of CPT from the micelles was observed to be higher than that of the free drug in both Caco-2 standard model (a) and a 3D intestinal model (b), with significant differences becoming evident during the final incubation periods. In the 3D model, CPT permeability reached approximately 27%, representing an enhancement compared to the standard model´s permeability of 20%. This heightened permeability in the 3D model aligns more closely with the in vivo human intestine, reflecting a drug permeability that is more akin to real-world conditions. The experiment also demonstrated consistent transepithelial electrical resistance (TEER) maintenance for both micelles and the free drug, reflecting the monolayer integrity and consequently suggesting that the tested formulation is safe. Besides, the apparent permeability coefficients exhibited notable disparities between the micelles and the free drug. In the 3D model, the apparent permeability coefficients of CPT from the micelles indicate a significant increase in CPT permeability and, consequently, bio-availability. On the other hand, the free drug maintained an apparent permeability coefficient that was similar in both models, signifying that the drug´s permeability was nearly half that of the micellar drug, specifically within the context of the 3D model (Figure 3D(c)). Furthermore, to ensure the structural integrity of the membrane following the permeability experiment, hematoxylin, and eosin (H&AND) staining was conducted for both models and under all conditions (Figure 3E). This staining process employed two dyes: hematoxylin, a basic dye, and eosin, an acidic dye. Hematoxylin imparts a purple hue to acidic structures, such as the nucleus, while eosin imparts a pink color to basic structures, such as the cytoplasm and extracellular matrix. Considering this staining mechanism, Figure 3E presents a consistent monolayer in both models, which underscores the preservation of cellular membrane integrity. These observations indicate that the membrane remained intact and well-formed throughout and following the permeability assay. Overall, this study’s findings showed that the printfills were able to keep the micelles intact until they reached the intestinal pH, increasing the CPT intestinal absorption and, consequently, its oral availability. Furthermore, the combination of 3D printing and nanotechnology holds considerable potential for the targeted release of polymeric micelles in the colon. This advancement can enhance the absorption of drugs in the intestines while safeguarding them from degradation as they go through the gastrointestinal tract [77].

In another study, researchers focused on creating a bio-ink suitable for 3D printing a hydrogel implant with controlled drug release capability. To achieve this, simvastatin was loaded into polymeric micelles composed of polylactide/poly (ethylene glycol) triblock copolymers (PLA-PEG-PLA). These micelles were then incorporated into hydrogels via a photo-cross-linking 3D printing process. The resulting simvastatin-loaded triple-network hydrogel demonstrated remarkably long-term drug release for over 14 weeks, consistently maintaining a therapeutic concentration. These findings indicate that these micelles hold great promise as a bio-ink material, providing long-term hydrogel stability, biodegradability, and sustained delivery of hydrophobic drugs, such as simvastatin [79].

#### 4.1.2. Liposomes/Niosomes

Due to their numerous benefits, liposomes have garnered significant attention, especially in the fields of cancer treatment and vaccinology. However, their development remains a challenge, making it crucial to seek out innovative approaches that enable fast, safe, and consistent production with high-level batch-to-batch reliability [80].

In an experimental study, researchers used 3D printing technology to create a 3D-printed niosomal hydrogel (3DP-NH) containing cryptotanshinome (CPT) as a topical delivery system for acne therapy. To formulate the CPT-loaded niosomal hydrogel, the CPT-loaded niosomes were carefully added, drop by drop, into the hydrogel. Subsequently, the resulting mixture was printed using an extrusion-based 3D printer to produce a 3D-printed CPT-loaded niosomal hydrogel (3DP-CPT-NH) with a specific drug dosage, shape, and size. The findings were that the 3DP-CPT-NH exhibited a significant anti-acne effect without causing any skin irritation [81].

Another investigation was conducted, combining microfluidic technology (MF) and 3D printing, leading to the formulation of “diamond-shaped” devices designed for the production of liposomes loaded with lysozyme as a model drug. Computer-aided design software was used to design microfluidic devices with diverse geometries, which were then printed using high-resolution digital light processing (DLP)—3DP. Stability tests confirmed the consistency of the developed formulations, and an encapsulation efficacy study showed positive results. Overall, this study showcased the effectiveness of combining MF and 3DP, highlighting the potential for synergistic growth in this field [80].

#### 4.1.3. Nanocrystals

In the pharmaceutical field, 3D printing has also been making an impact in the manufacturing of nanocrystals, allowing for more precise control over drug dosage and release profiles [82]. For instance, additive manufacturing has been used to encapsulate nanocrystals within polymeric matrices, creating drug-loaded filaments or tablets, and improving the solubility and bio-availability of poorly soluble drugs. The precise control over the placement of nanocrystals in the printed structure allows for enhanced drug delivery and therapeutic efficacy. In fact, a recent study aimed to develop fast-dissolving oral polymeric film formulations loaded with indomethacin nanocrystals using 3D printing technology, and the outcomes demonstrated that this offers a promising approach for enhancing solubility in immediate-release formulations [83].

Another experiment focused on the development of an in situ forming robust injectable and 3D printable hydrogel based on cellulose nanocrystals. The results demonstrated that the hydrogels exhibited excellent injectability and maintained their shape fidelity without the need for additional cross-linking steps. The interlayer bonding between the printed layers was strong, resulting in the formation of sTable 3D structures, even up to 10 layers [84].

Additive manufacturing also allows for the creation of personalized drug delivery systems by incorporating nanocrystals. For instance, researchers have used 3D printing to fabricate patient-specific tablets containing nanocrystals of poorly soluble drugs. This enables customized dosages and controlled release profiles to be tailored to individual patient needs. In the first-ever study that included nanocrystals within 3D-printed tablets, albendazole nanocrystals were successfully incorporated into tablets, achieving a concentration of up to 50% *w*/*w*, which is not typically attainable with conventional tablets. Moreover, the printlet formulation with nanocrystals exhibited superior efficacy in improving drug dissolution in HCL 0.1N when compared to nanocrystals in hard gelatin capsules. The nanocrystals exhibited consistent particle size, crystallinity, and chemical stability both before and after 180 days of storage. Overall, the findings demonstrated the promising pharmaceutical potential of combining 3D printing and nanocrystals for the development of stable, fast-release, oral solid dosage forms of poorly soluble drugs. This experiment employed propylene glycol as a carrier and demonstrated that this technique holds promise for printing objects utilizing various types of nanocrystals embedded in low-melting-temperature polymers [85].

### 4.2. In Silico Modeling

#### 4.2.1. Polymeric Micelles

**Figure 4:**
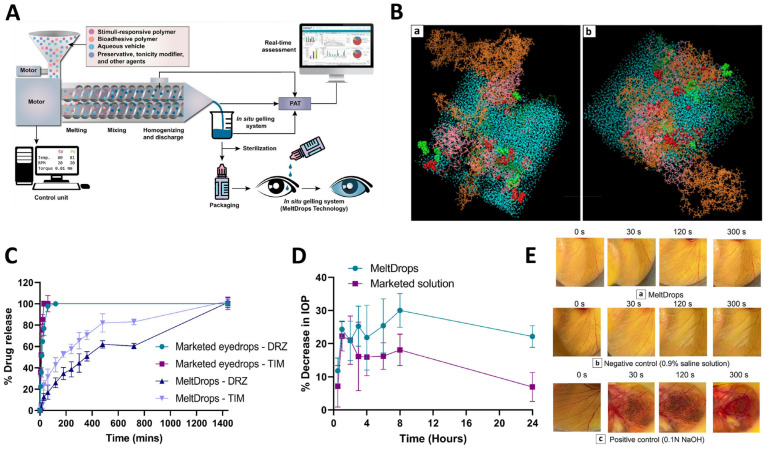
(**A**) Schematic depiction of the ISGS manufacturing process via MeltDrops technology. (**B**) Molecular interactions forming between the components of the formulation at two different temperatures: 300 K (**a**) and 308 K (**b**): Green CPK model = TIM; Red = DRZ; Blue = Sodium chloride; Yellow = Benzalkonium chloride; Pink = HPMC; Orange = Poloxamer 407; Green wire model = Poloxamer 188; Blue = water. (**C**) Cumulative release of timolol maleate (TIM) and dorzolamide hydrochloride (DRZ) over time from both MeltDrops and commercially available solutions. (**D**) Percentage decrease in IOP following the administration of MeltDrops and commercially available eyedrops. (**E**) HET-CAM test results after the application of (**a**) MeltDrops with no signs of irritation, (**b**) 0.9% *w*/*v* saline solutions, also showing no signs of irritation, and (**c**) 0.1 N Sodium hydroxide solution, revealing features such as vascular lysis, coagulation, and hemorrhage [86]. **DRZ**: dorzolamide hydrochloride; **IOP**: intraocular pressure; **ISGS**: in situ gelling system; **TIM**: timolol maleate.

A recent study focused on developing a novel technology called MeltDrops, which used hot-melt extrusion (HME) for continuous manufacturing of in situ gelling systems (ISGS) known to prolong the retention time and improve the bio-availability of ophthalmic drugs. This is relevant because the traditional manufacturing of ISGS has been challenging and costly, hindering their industrial scale-up and clinical implementation. However, MeltDrops technology offers a one-step extrusion process to develop these systems (Figure 4A), which overcomes the limitations of batch manufacturing. Based on in silico modeling, researchers employed a molecular dynamics (MD) simulation to analyze the difference in physical properties of two types of MeltDrops—loaded with timolol maleate (TIM) or dorzolamide hydrochloride (DRZ)—under two different temperature conditions, 300 K (room temperature) and 308 K (physiological temperature) (Figure 4B). These simulations offered evidence of heightened interactions among drug, polymer, and water molecules at a higher temperature (308 K), suggesting the formation of ISGS with desired properties, including a solution–gel transition at physiological temperatures. Researchers also concluded that the in vitro drug release from MeltDrops technology demonstrated sustained and controlled release behavior, while marketed eyedrops showed complete drug release in less than 30 min (Figure 4C). Besides, the results demonstrated a percentage decrease in intraocular pressure (IOP) following the administration of MeltDrops and marketed eyedrops, highlighting the superior IOP-reducing potential of MeltDrops compared to conventional options (Figure 4D). Finally, a HET-CAM test was conducted in order to evaluate the potential ocular irritancy of MeltDrops. The results showed no signs of irritation, indicating that they are safe and well tolerated for ocular use [86].

Previously, a continuous manufacturing technique that utilizes a coaxial turbulent jet in co-flow was established for the production of paclitaxel-loaded polymeric micelles. More recently, researchers have employed coarse-grained molecular dynamics simulations to gain deeper insights into the impact of material attributes (specifically, the drug-polymer ratio and ethanol concentration) and process parameters (such as temperature) on the self-assembly process of polymeric micelles. Additionally, these simulations provided molecular-level information on micelle instability. The findings demonstrated a clear correlation between the micelle shape and drug encapsulation. As the paclitaxel content increased, the micelles transformed from spherical to ellipsoidal structures. From the simulation data, researchers were able to identify the critical aggregation number, which represents the minimum number of polymer and drug molecules required for this shape transition. Moreover, this investigation indicated that larger micellar size and reduced solvent accessibility contributed to the enhanced structural stability of the micelles. Additionally, researchers conducted an evaluation of the micellar dissociation free energy using steered molecular dynamics simulations across various temperatures and ethanol concentrations. The simulations showed that higher ethanol levels and temperatures led to micellar destabilization, resulting in a more significant release of paclitaxel. This increased drug release was attributed to the solvation of the hydrophobic core, promoting micellar swelling and reducing hydrophobic interactions, ultimately leading to a loosely packed micellar structure. In general, the computational predictions provided valuable insights into the micelle self-assembly process, morphological changes, drug release, and thermodynamic instability and showed excellent agreement with experimental results, underscoring its efficacy in studying the impact of material attributes and process parameters on the polymeric micelle formulation during continuous processing [87].

In another study, researchers used coarse-grained molecular dynamics simulations to investigate the behavior of a specific type of block copolymer called poly (ethylene oxide)-poly (propylene oxide)-poly (ethylene oxide) (PEO-PPO-PEO), commonly known as pluronics or poloxamers. They studied the effect of polymer and surfactant concentration on the morphology of these block copolymers and ionic surfactants, namely sodium dodecyl sulfate (SDS), in aqueous solutions. The results showed that when pluronics and SDS are present together in the solution, they tend to form mixed micelles and that the shape of those micelles depends on the relative concentrations of pluronics and SDS in the solution. The core of the mixed micelles consists of PPO chains from the pluronics, the alkyl tail of SDS, and some water molecules. The surrounding shell is composed of PEO chains, water molecules, and the sulfate headgroups of the SDS. Notably, with an increasing amount of added SDS, the observed morphology of the mixed micelles undergoes a transition from spherical to wormlike–cylindrical geometry. Overall, the molecular insights gained from studying the co-assembly of an ionic surfactant and an amphiphilic triblock copolymer in aqueous media have potential applications in various complex fluid mixtures. However, the accuracy of the results relies on the coarse-grained force field used, which can be improved with more computationally expensive atomistic simulations for quantitative comparisons with experimental data [88].

#### 4.2.2. Liposomes/Transfersomes

In silico modeling has been employed in the manufacturing of liposomes because it allows researchers to simulate and predict the behavior of these structures, such as their stability, size, composition, drug encapsulation efficiency, and release kinetics. Furthermore, these models can help to optimize the manufacturing process, predict the performance of liposomal formulations, and guide experimental design [89].

In a recent study, the authors constructed computational models to identify active pharmaceutical ingredients (APIs) that can achieve the desired high concentrations in nano-liposomes via remote loading. The models aimed to predict the suitability of APIs for nano-liposomal delivery by considering fixed main experimental conditions, such as liposome lipid composition and size. The researchers also added a prediction of drug leakage from the nano-liposomes during storage, which is crucial for ensuring the development of pharmaceutically viable nano-drugs. More so, by using “load and leak” models, this group screened two large molecular databases to identify candidates’ APIs for delivery by nano-liposomes. Through the screening process, the researchers identified 667 molecules that showed positive results in both the loading and leakage models, indicating high-loading and stable characteristics. Among these molecules, 318 received high scores in both properties and, notably 67 of them are FDA-approved drugs [90]. These findings underscore the significance of computational modeling in optimizing liposomal formulations. By narrowing the search to molecules exhibiting high-loading and stability characteristics, researchers can concentrate their efforts on candidates with a higher likelihood of success. This approach facilitates efficient use of time, resources, and cost savings compared to traditional trial-and-error methods.

These computational approaches can—and should—be combined with experimental studies because it allows for a better understanding of the mechanisms that are being investigated. Computational modeling not only aids in explaining experimental results, but has also the potential to guide and inspire new directions for experimental research in the development of liposomal drug delivery systems [91].

In a study that aimed to develop active targeting liposomes to deliver anticancer agents to the treatment of hepatocellular carcinoma, computational modeling was used to gain insight into the structure and behavior of the intended targeted liposomal drug delivery systems within the bloodstream. This research showcases the complementary nature of these simulations alongside experimental research, often offering valuable mechanistic context [92].

Another example of the predictive power of in silico modeling in the pharmaceutical industry is an investigation that explored the use of thermosensitive liposomes (TSL) for targeted drug delivery to tumors. The researchers created a three-dimensional computer model to simulate the delivery of the TSL-encapsulated doxorubicin to mouse tumors. To do so, a mouse hind limb was scanned using a 3D scanner, and the resulting geometry was imported into finite element modeling software. A virtual tumor was added to the model, and the authors simulated the heating process using a surface probe. In addition to the heat transfer model, the researchers also developed a drug delivery model that simulates the kinetics of drug release. It is important to mention that the computed model was validated by performing experimental studies using gel phantoms and in vivo fluorescence imaging studies in mice with lung tumor xenografts. By comparing the results of the computer model with the experimental studies, the researchers can assess the accuracy of the model. The results showed that in silico modeling accurately reproduces the temperature profile observed in the phantom experiments and that the drug delivery profile simulated by the model also aligns with the results of the in vivo studies. Overall, it demonstrates the feasibility of using a computer model to accurately simulate drug delivery in preclinical studies [89].

To investigate the distribution of three drugs with different polarities (5-fluorouracil, ligustrazine, and osthole) within liposomes and transfersomes, researchers conducted a study using molecular dynamics simulation. To understand the drug distribution, these authors employed the radial distribution function—which calculates the probability of finding a drug molecule at a specific distance from a reference drug molecule within the vesicle—and the potential of mean force—which describes the potential energy between a drug molecule and the surrounding lipid molecules, indicating the strength of their interactions. By using these measures, the authors were able to characterize the distribution of drugs within the lipid vesicles. The results highlight the potential of molecular simulation technology in understanding the characteristics of lipid vesicles and their interactions with drugs [93].

#### 4.2.3. Nanocrystals

A study aimed to develop and evaluate an advanced in silico modeling for understanding the pharmacokinetics of Foscan^®^, a formulation containing temoporfin that has received approval for palliative photodynamic therapy of squamous cell carcinoma of the head and neck. The researchers conducted precipitation experiments in the presence of biorelevant media, thereby simulating conditions akin to those encountered in the human body. This approach aimed to observe the behavior of Foscan^®^ under these physiologically relevant circumstances. When introduced in these media, the drug underwent a process of precipitation, forming nanocrystals. Moreover, nanoparticle tracking analysis was employed to investigate these nanocrystals, providing the means to measure their size and analyze the distribution of these structures within the sample. Incorporating the data from these precipitation experiments and nanoparticle tracking analysis, the model predicted how nanocrystals of Foscan^®^ were formed, their size distribution, and how they interacted with biological fluids in the body. This information could help them explain and predict the Foscan^®^ pharmacokinetics more accurately, as nanocrystals can significantly impact how a drug is absorbed, distributed, and eliminated [94].

In another study, to evaluate the impact of polymers in the production of stable dexibuprofen (Dexi) nanocrystals with improved therapeutic potential, researchers combined in silico modeling techniques (namely AutoDockVina, Marven Sketch, and Maestro) with experimental studies. The results provided molecular insight into the mechanisms of binding of the optimal polymers to the surface of Dexi nanocrystals, showing that the combination of hydroxypropyl methylcellulose (HPMC)-polyvinyl pyrrolidone (PVP) and HPMC-Eudragit (EUD) was the most effective in stabilizing Dexi nanocrystals. Overall, the combination of computational modeling with experimental studies allows researchers to save time and resources by focusing on the most promising polymer combinations, thereby expediting the drug development process. Additionally, this integrated approach provides a deeper understanding of the molecular mechanisms underlying the stabilization of nanocrystals, helping researchers make more informed decisions in their pursuit of developing better pharmaceutical formulations [95].

### 4.3. Machine Learning

#### 4.3.1. Polymeric Micelles

Machine learning algorithms can be utilized to predict various properties and behaviors of polymeric micelles. For example, models can be trained using data on polymer structure, composition, molecular weight, and other relevant parameters, along with experimental outcomes such as micelle size, stability, drug loading, and release profiles. These models can then be used to predict the behavior of new polymeric systems, guiding the design and selection of optimal micellar formulations [96].

Researchers used an artificial neural network (ANN) to create a model for the release of a chemotherapeutic drug—doxorubicin—from polymeric micelles (specifically Pluronic P105) under two different ultrasound frequencies. Although the exact number of samples used in the study was not explicitly mentioned, the model was trained using experimentally obtained input–output data concerning the release of doxorubicin from the micelles. The developed ANN model was then employed to optimize the application of ultrasound in order to achieve the desired drug release at the tumor site. The ANN method accurately predicted the release behavior and demonstrated maximum prediction errors of 0.002 and 0.001 at ultrasound frequencies of 20 and 70 kHz, respectively. The results demonstrate the successful design and testing of a controller capable of adjusting ultrasound frequency, intensity, and pulse length to maintain a constant release of Dox, potentially enhancing targeted drug delivery to tumor sites [97].

#### 4.3.2. Liposomes/Niosomes

By analyzing large datasets of liposomal properties and characteristics, machine learning models can identify patterns and correlations between various liposome components (lipids and encapsulated substances, among others) and their properties (size, stability, drug release profile). This information can guide the selection of optimal liposome compositions and improve formulation success rates [98]. In addition, ML models can also be trained on existing data to predict important liposomes properties, which may include encapsulation efficiency, drug release kinetics, stability under different conditions, and targeting capabilities. By utilizing historical data and relevant features, machine learning algorithms can provide valuable insights and predictions, enabling more efficient and targeted liposome development [99].

In fact, there are some trials applying machine learning for liposome formulation optimization or prediction. For instance, one study proposed a machine learning framework to address the challenges associated with optimizing the drug entrapment efficiency of niosomal vesicles, showing that these algorithms allow for the synthesis of niosomal systems with optimal entrapment efficiency at a lower cost and time (Kashani-Asadi-Jafari et al., 2022). In another study, scientists built an artificial neural network (ANN) and advanced machine learning model to optimize the percentage of cytarabine entrapped in the liposome, showing that the ANN provided more accurate prediction formulations when compared with the multiple regression analysis method [100]. An ANN model was also developed to predict the size and polydispersity index of liposomes made of DOPC (1,2 Dioleoyl-sn-glycero-3-phosphocholine), cholesterol, and DSPE-PEG 2000 1,2 Distearoyl-sn-glycero-3-phosphoethanolamine-N [amino (polyethylene glycol)-2000] (ammonium salt)) using a microfluidic system. The results demonstrated that microfluidic-based preparation techniques assisted by computational tools can accelerate the development and clinical translation of nano-based pharmaceutical products [101].

**Figure 5:**
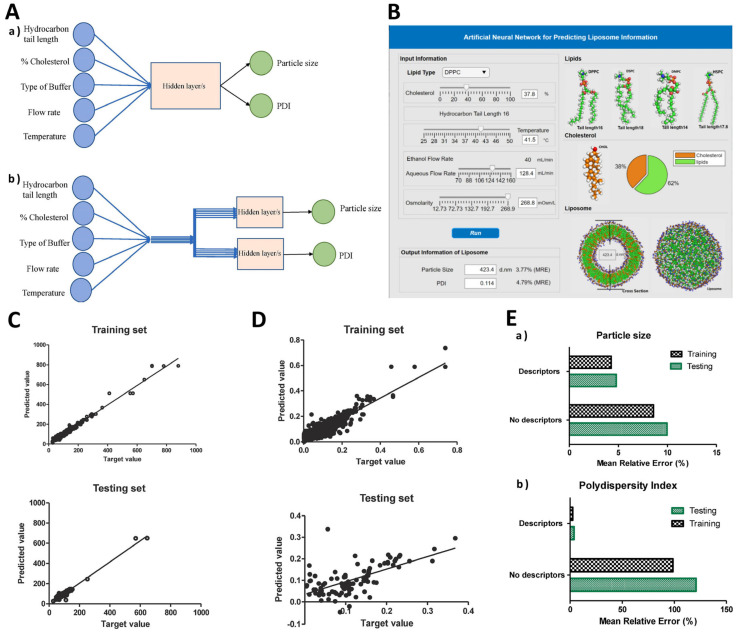
(**A**) Comparative illustration of MIMO model (**a**) and MISO model (**b**). (**B**) Graphical user interface of the ANN for liposome particle size and PDI prediction in continuous liposome manufacturing. (**C**) Comparison of predicted vs. target values for liposome particle size in the training and testing sets, without molecular descriptors. (**D**) Comparison of predicted vs. target values for liposome PDI in the training and testing sets, without molecular descriptors. (**E**) Evaluation of MRE with and without incorporating molecular descriptors in the ANN input: (**a**) particle size and (**b**) PDI [103]. **ANN**: artificial neural network; **MIMO**: multiple-input–multiple-output; **MISO**: multiple-input–single-output; **PDI**: polydispersity index; **MRE**: mean relative error.

Recently, machine learning has been combined with molecular descriptors, which are a set of quantitative values or features that represent various properties of a molecule’s structure, composition, and behavior. These are used to encode complex chemical information into numerical data, which can then be used as input for various computational analysis and machine learning models. Fundamentally, ML models leverage patterns within data to predict the properties of novel molecules, eliminating the need for physical synthesis or testing [98,102]. To illustrate this, an ANN was constructed to develop computational models focused on optimizing a continuous liposome manufacturing system. In this system, the liposomes were generated using a co-axial turbulent jet within a co-flow technology. This means that two phases were used—an ethanol phase with lipids and an aqueous phase—to create liposomes of uniform sizes. The ANN was used to optimize this manufacturing process and so, it took various input parameters known as critical material attributes (CMAs) and critical process parameters (CPPs). CMAs include characteristics of the raw materials, such as the length of the hydrocarbon tail in lipids, the percentage of cholesterol, and the type of buffer used. CPPs include process conditions, such as solvent temperature and flow rate. The ANN’s purpose was to predict critical quality attributes (CQAs) of the liposomes. In this study, the CQAs were the particle size and polydispersity index (PDI), which indicate how uniform the liposome sizes are. Thus, two types of ANN architectures were evaluated, namely a multiple-input–multiple-output (MIMO) model—which takes multiple inputs and produces multiple outputs—and a multiple-input–single-output (MISO) model—which takes multiple inputs but produces a single output (Figure 5A). The study found that the MISO architecture outperformed the MIMO architecture in terms of accuracy for the task at hand. Apart from the ANN model, a graphical user interface was also created to help end-users perform interactive simulated risk analysis and visualize the predictions made by the ANN model (Figure 5B). Evaluations demonstrated that the developed graphical user interface yields accurate predictions for both liposome particle size and PDI as long as the chosen inputs fall within the scope of the studied conditions during the initial ANN training. These predictions have the potential to contribute to the formulation of a control strategy designed to mitigate the impact of process disturbances on liposome particle size. Utilizing the five input features mentioned earlier, an ANN was trained with the primary goal of minimizing the mean relative error (MRE), which was successfully achieved at a level below 5%. It was notably a very low error for predicting particle size, as is evident from the comparison between the target and predicted values presented in Figure 5C. However, the prediction accuracy for PDI was notably inadequate, as indicated by the results displayed in Figure 5D. Basically, despite the successful predictions for particle size, the model encountered challenges in accurately forecasting PDI values. To mitigate the training error, researchers introduced molecular descriptors as supplementary inputs to the ANN. These were obtained using PaDEL-Descriptor software and helped the ANN understand the characteristics of the raw materials. A combination of CMAs, CPPs, and molecular descriptors was used to train the MISO ANN model and allowed for the reduction of errors during both training and testing, indicating improved model performance (Figure 5E). Overall, via a combination of critical material attributes, process parameters, and molecular descriptors, this study improved the accuracy of predicting the quality attributes of liposomes [103].

**Figure 6:**
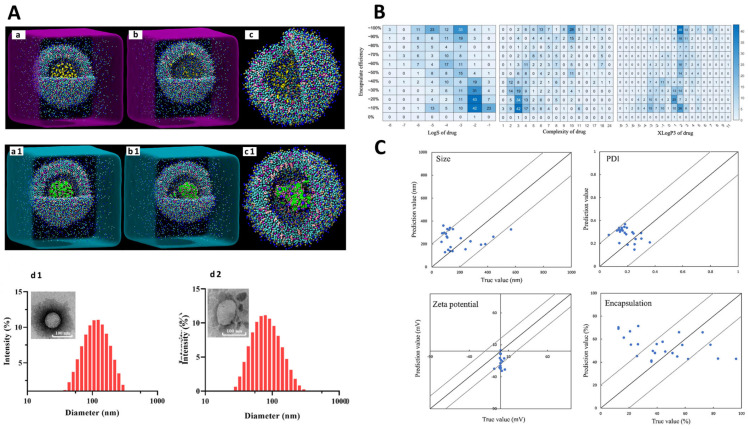
(**A**) Snapshots captured during 1 microsecond of molecular dynamics simulations, alongside the experimental size distribution of NAP liposomes (**a**–**c**,**d1**) and PAL liposomes (**a1**–**c1**,**d2**). (**B**) Heat map assessment illustrating the connection between logS, molecular complexity, and XLogP3 of the drug with encapsulation within liposomes. The numerical values within each matrix indicate the frequency of encapsulation occurrences corresponding to each specific property. (**C**) A scatter plot displaying the comparison between experimental and predicted values for size, PDI, zeta potential, and encapsulation [98]. **NAP**: naproxen; **PAL**: palmatine HCL; **PDI**: polydispersity index.

In another study, ML techniques were used to create prediction models capable of individually predicting crucial parameters of liposomes, such as size, PDI, zeta potential, and encapsulation efficiency. To validate the predictive prowess of these models, liposome formulations were created for two distinct compounds: naproxen (NAP) and palmatine HCL (PAL), representing insoluble and water-soluble molecules, respectively. In order to evaluate the significance of drug properties in liposome behavior, further investigation into the molecular interactions and behaviors of NAP and PAL within liposomes was undertaken via coarse-grained molecular dynamics simulations. Figure 6A(a,a1) depict the initial configurations of the two systems, of which ten underwent dynamic simulations lasting 1 microsecond. Snapshots captured during the modeling process are illustrated in Figure 6A(b,b1), while the ultimate structures of the respective liposomes are displayed in Figure 6A(c,c1). Additionally, the size distribution was assessed, and the structures of the liposomes containing NAP and PAL were characterized using transmission electron microscopy, showcased in Figure 6A(d1,d2). These simulations demonstrated that NAP molecules tend to integrate into the lipid layer, while a majority of PAL molecules aggregate within the inner aqueous phase of the liposome. The marked disparity in the physical states of NAP and PAL underlines the pivotal role of drug properties in formulating liposomes. Additionally, formulation attributes were ranked to offer significant insights for designing effective formulations. Given that logS (logarithm of a compound’s aqueous solubility), molecular complexity (an assessment of the intricacy of a structure), and XLogP3 (represent a predictive estimation of the octanol–water partition coefficient, determined via a specific algorithm) of the drug molecules held significant influence over encapsulation efficiency, their correlation was illustrated using a heatmap, depicted in Figure 6B. This heatmap employed color visualization in a two-dimensional format to depict the data relationship effectively. Basically, drug molecules with certain properties, such as a logS value between −3 and −6, a molecular complexity between 500 and 1000, and a XLogP3 value greater than or equal to 2, are considered a priority for formulating liposomes with better encapsulation. Finally, in Figure 6C, it is possible to observe a congruence between predicted and experimental outcomes, which serves as confirmation of the ML model’s satisfactory accuracy. In summary, the researchers established comprehensive prediction models for anticipating liposome formulations, and the influences of key factors were dissected by combining ML techniques with molecular modeling. The study successfully validates the availability and rationality of these intelligent prediction systems, offering promising applications for the future development of liposome formulations [98].

Based on all of that, it is safe to say that machine learning plays a valuable role in the development of liposomes by assisting in formulation design, predicting liposome properties, optimizing drug loading and release, analyzing characterization data, and optimizing manufacturing processes. By leveraging machine learning techniques, researchers can expedite the development and improve the performance of liposomal formulations for drug delivery and other biomedical applications [104].

#### 4.3.3. Nanocrystals

Machine learning techniques have been increasingly employed in the field of nanocrystal development. For instance, they can be trained to predict the properties of nanocrystals by using data from a variety of sources, including experimental measurements, theoretical calculations, and molecular descriptors. These predictive models can assist in the design and selection of nanocrystals with desired properties, saving time and resources by reducing the need for extensive experimental testing. In fact, to address this issue, researchers collected data on nanocrystal size (910 data points) and polymer dispersity index (341 data points) using three different preparation methods—ball wet milling (BWM), high-pressure homogenization (HPH), and antisolvent precipitation (ASP)—in order to construct prediction models [105].

**Figure 7:**
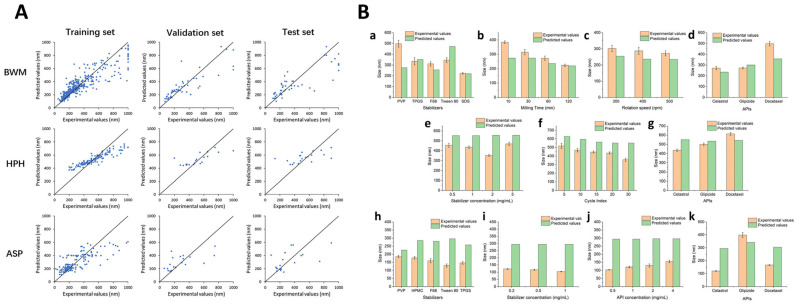
(**A**) Scatter plots comparing the predicted values obtained from the machine learning algorithm with the experimental values for nanocrystal size on the training, validation, and test subsets within the BWM, HPH, and ASP size datasets. (**B**) Contrasts in the predicted values generated by the algorithm with the actual sizes of nanocrystals prepared with BWM (**a**–**d**), HPH (**e**–**g**), and ASP (**h**–**k**) [105]. **ASP**: antisolvent precipitation; **BWM**: ball wet milling; **HPH**: high-pressure homogenization.

The results indicated that the machine learning performed well in predicting those properties for BWM and HPH methods but showed relatively poor predictions for the ASP method. Figure 7A displays scatter plots comparing the predicted values, generated by the machine learning model, with the experimental values of nanocrystals sizes within BWM, HPM, and ASP size dataset subsets. Within the BWM subsets, the predicted values closely matched the experimental values in the size range of 0–500 nm. Nevertheless, for data points outside of this range, particularly those exceeding 500 nm, predicted values displayed significant disparities from the experimental values, especially in the validation and test datasets. The findings suggest that the constructed learning model exhibits superior predictive accuracy for data points falling within the 0–500 nm range, particularly where the data density is higher in the BWM size dataset. It is evident that the uneven distribution of data within the dataset significantly influences the model’s construction and predictive performance. On the other hand, due to the limited availability of input data within the size range of 500 to 1000 nm, learning algorithms struggled to discern the underlying data patterns, leading to less accurate predictions. The scatter plots for the HPH and ASP subsets showed comparable results, with predicted values closely aligning with experimental values in regions where data density was higher [105].

**Figure 8:**
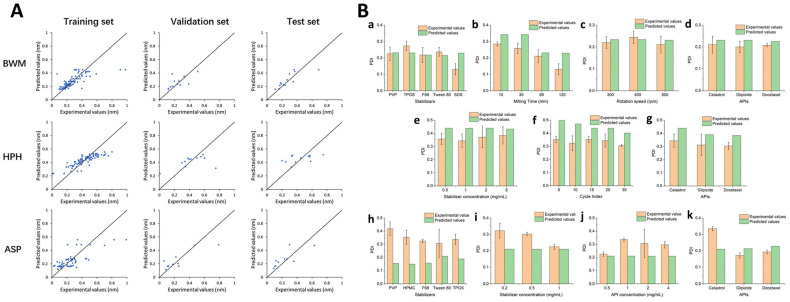
(**A**) Scatter plots comparing the predicted values obtained from the machine learning algorithm with the experimental values for nanocrystal PDI on the training, validation, and test subsets within the BWM, HPH, and ASP size datasets. (**B**) Contrast the predicted values generated by the algorithm with the actual PDI values of nanocrystals prepared by BWM (**a**–**d**), HPH (**e**–**g**), and ASP (**h**–**k**) [105]. **ASP**: antisolvent precipitation; **BWM**: ball wet milling; **HPH**: high-pressure homogenization; **PDI**: polydispersity index.

Figure 8A, on the other hand, displays scatter plots comparing the predicted values to the experimental values of PDI data. The machine learning algorithm performed admirably in predicting this property values within the BWM and HPH subsets, as indicated by the close alignment of data points with the black line. Despite the relatively small datasets with only 133 and 119 data points in the BWM and HPH PDI datasets, respectively, the algorithm still demonstrated accurate predictions, likely due to the concentrated data distribution. Conversely, in the ASP subsets, the predictive performance in the training set was notably less accurate. This could be attributed to the smaller volume of data and the lower data quality within these subsets [105].

The researchers speculated that the poor prediction for the ASP method might be due to the lower quality of data resulting from the poor reproducibility and instability of nanocrystals prepared using this method. It was also found that the majority of commercialized nanocrystals products were manufactured using BWM and HPH approaches. ML helped rank the factors influencing nanocrystal properties, indicating that milling time, cycle index, and stabilizer concentration were crucial factors for the nanocrystals prepared by BWM, HPH, and ASP methods, respectively. The accuracy of these predictions was further confirmed by experiments with newly prepared nanocrystals. Concerning the nanocrystals size, the findings indicate that the algorithm delivered accurate predictions for most of the nanocrystals produced through the BWM (Figure 7B(a–d)) and HPH (Figure 7B(e–g)) methods. However, the predicted performance for ASP nanocrystals (Figure 7B(h–k)) fell short, with predicted values being roughly twice as large as the experimentally measured sizes. Regarding PDI predictions, the algorithm also demonstrated effective forecasting for BWM (Figure 8B(a–d)) and HPH (Figure 8B(e–g)) datasets, but showed comparatively poorer performance for ASP (Figure 8B(h–k)). The most likely reasons for this subpar predictive performance were the limited volume of data available for ASP size and PDI datasets used in constructing machine learning models, and the lower data quality stemming from the constraints of the preparation methods. These issues ultimately led to the failure of predictions within the ASP dataset.

Overall, the results highlights the potential of using machine learning in nanotechnology manufacturing, providing a promising alternative to traditional, labor-intensive approaches in nanocrystal formulation development [105].

### 4.4. Digital Twins

Digital twins have emerged as a transformative technology in the pharmaceutical industry, offering a powerful tool for improving drug development processes and product lifecycle management [106]. In the realm of traditional pharmaceuticals, their significance is already well recognized, as digital twins enable the creation of virtual replicas of physical drugs and manufacturing processes, facilitating real-time monitoring, optimization, and quality control [39,40]. However, it is important to highlight the lack of experimental studies on the application of these technologies in the NBCD domain. This is an exciting frontier for innovation and has the potential to significantly advance the development, manufacturing, and quality assurance of these pharmaceutical products.

## 5. Regulatory Issues

Regulatory agencies, including the FDA, believe that the incorporation of computational methods into the field of pharmaceutics can enhance product quality. When these methods are used, a deeper understanding of the process involved in product design is achieved, aligning with the principles of QbD [7]. In fact, the FDA has been moving towards performance-based regulation, focusing on measurable outcomes rather than prescriptive processes, which aligns well with Pharma 4.0 and its extensive data capabilities [3].

To attain Pharma 4.0, it is essential to embrace cutting-edge manufacturing technologies while simultaneously surmounting regulatory obstacles [12]. For instance, initially, the absence of a well-defined regulatory framework was notable, hindering innovators from integrating digital technologies with traditional processes due to a lack of regulatory precedents. However, more recently, significant strides have been taken in this direction. In particular, the FDA has taken a proactive step by establishing the Digital Health Center of Excellence, aiming to adopt a comprehensive approach to digital health technology [107].

Moreover, filing regulatory applications across different jurisdictions with varying expectations can be burdensome, particularly for emerging manufacturing technologies. Achieving international regulatory convergence could provide clarity and certainty for manufacturers. Besides, the industry may simultaneously comprise companies operating under Pharma 2.0, 3.0, and 4.0 paradigms. Regulating such a diverse landscape requires flexible frameworks to enable the adoption of new technologies without disrupting supply from older technology-based manufacturers [3].

It is important to note that, at present, the FDA has already approved several 3D printed products, which encompass not only drug products—such as Spritam^®^—but also—and essentially—a diverse range of medical devices that include orthodontic implants, prosthetics, anatomical models, and reaction-wares [108]. In December 2017, the FDA released a set of guidelines titled “Technical Considerations for Additive Manufactured Medical Devices”, which provides guidelines covering various aspects, including software and hardware requirements, quality control, and process validation procedures. However, considering the complexity of medicinal products, which often have more demanding requirements than medical devices, separate regulatory considerations are needed. APIs present additional challenges, such as potential incompatibilities and the stability of the active substance during the printing process [109].

Nonetheless, due to the variability of additive manufacturing methods, it is challenging to devise a universal set of guidelines applicable to all 3D printing techniques. The truth is that, given the multitude of factors influencing the quality of computationally designed dosage forms and the safety of their use, the establishment of appropriate regulatory requirements is of utmost importance. However, currently, there are no valid regulations concerning the design, manufacturing process, and quality testing considerations specific to three-dimensional printing in the pharmaceutical industry [110]. Recently, it has been emphasized that 3D printing, as a manufacturing process, does not pose regulatory limitations as long as the final product meets the established requirements. For instance, the previously mentioned approved 3D printed tablet, Spritam^®^, includes the same excipients that are found in conventional tablets. The only difference lies in the production process. Consequently, it is reasonable to assume that similar quality requirements apply in this case as they do for other orally disintegrating tablets [111].

Overall, as the pharmaceutical industry is transitioning into the Pharma 4.0 era, regulatory agencies are exploring ways to adapt and accommodate the advancements brought by these new technologies [107].

## 6. Future Prospects

Pharma 4.0 technologies have demonstrated significant promise in the manufacturing of NBCDs. The integration of advanced digital techniques, automation, and data analytics in the pharmaceutical industry has the potential to optimize processes, enhance quality control, enable customization, and improve supply chain efficiency, ultimately leading to improved production and delivery of NBCDS.

However, there is still much potential for further development and some areas where ongoing work and future efforts can be focused. For instance, these technologies can extend to various other NBCDs, such as glatiramer acetate and iron carbohydrate complexes as, to the best of our knowledge, there are no experimental studies available that assess the impact of Pharma 4.0 in these NBCDs. The implementation of digital twins is also expected to assist in simulating and optimizing NBCD manufacturing. By integrating real-time data from sensors and process models, digital twins can enable virtual testing, scenario analysis, and optimization of manufacturing operations to enhance productivity and efficiency. It is important to take into consideration that as the implementation of Pharma 4.0 techniques progresses, regulatory frameworks need to evolve to accommodate the use of advanced technologies in NBCD manufacturing. This includes addressing data security and privacy concerns and establishing guidelines for the validation and qualification of digital systems while ensuring compliance with evolving regulatory requirements.

In the machine learning domain, SVMs and random forests stand out as increasingly promising alternatives to ANNs. Particularly in cases where datasets are limited, ANNs tend to be prone to overfitting due to their complex nature. SVMs, with their remarkable ability to capture intricate relationships within data, and random forests, with their ensemble learning capabilities, offer a more robust and adaptable solution for the challenges unique to NBCDs. These methods, by effectively handling complex NBCD compositions and diverse characteristics, could provide a brighter prospect for the domain, emphasizing precision, reliability, and the capacity to meet quality and safety standards [37,38].

Still in the machine learning domain, experimental studies that employ multiple metrics to quantify the model’s performance are surprisingly scarce. This gap in the research landscape highlights the need for a more comprehensive approach to assessing the effectiveness of these models. By incorporating a variety of evaluation metrics, such as accuracy, precision, recall, F1 score, ROC-AUC (receiver operating characteristic–area under the curve), and others tailored to the specific problem, researchers can gain a more nuanced and holistic understanding of their model’s performance. This multifaceted evaluation approach is vital in ensuring a more accurate and reliable appraisal, ultimately leading to better-informed decision-making and higher-quality work within the realm of machine learning [112,113].

In addition, it is also important to keep in mind that the implementation of Pharma 4.0 principles and technologies implies a reevaluation and readjustment of economic policies and legal frameworks and the establishment of financial stability to accommodate these emerging techniques. This also calls for a transformation in the academic curriculum to facilitate the acquisition of necessary skills, upskill the workforce, and foster system-wide awareness [6].

Overall, the prospects of Pharma 4.0 in the manufacturing of NBCDs are very promising. The implementation of advanced digital technologies and data-driven approaches can optimize processes, improve quality assurance, enhance customization capabilities, and streamline supply chains, ultimately leading to more efficient and effective NBCD manufacturing.

While the concept of Pharma 4.0 continues to grow and its techniques are slowly applied, there is an ongoing discussion about the potential evolution of the pharmaceutical industry beyond the current Pharma 4.0 framework. In fact, the term Pharma 5.0 has already been mentioned. It represents a possible future stage in which the integration of advanced technologies and data-driven approaches in the pharmaceutical sector goes even further. Hence, the prospects are very ambitious, considering the potential of these tools, but also a challenge, in particular for the regulatory follow-up that will have to be given in this context.

## 7. Conclusions

Concerning non-biological complex drug manufacturing, Pharma 4.0 techniques such as machine learning, in silico modeling, and 3D printing allow for enhanced overall production efficiency by streamlining operations and reducing production time, ultimately leading to cost savings. Additionally, the increased automation and use of real-time data analytics have improved process monitoring and control, minimized the risk of errors, and ensured a higher level of product quality and consistency.

However, the integration of Pharma 4.0 in the manufacture of NBCDs is still limited, essentially restricted to nanocrystals, liposomes, and polymeric micelles. It would be of great interest to extend the investigation of these techniques to other NBCDs, such as glatiramer acetate and iron carbohydrate complexes, for example. On the other hand, in the realm of machine learning, where extensive research has been conducted, it would be relevant to approach some other algorithms instead of focusing solely on ANN. Another constraint in the machine learning domain is the fact that there is a scarcity of experimental studies that use multiple metrics to gauge the model’s performance.

Digital twins seem to be very promising in the field of pharmaceutical industry, but they have not been applied to the domain of NBCDs. However, by integrating real-time data from sensors and process models, digital twins can enable virtual testing, scenario analysis, and optimization of manufacturing operations to enhance productivity and efficiency.

It is also important to acknowledge the challenges associated with the adoption of these technologies, such as the need for skilled personnel capable of operating and maintaining advanced technologies, ensuring data security and privacy, and addressing regulatory concerns regarding the validation and qualification of these novel manufacturing processes.

Overall, it is safe to say that the implementation of Pharma 4.0 technologies in the manufacturing of NBCDs represents a transformative approach and a paradigm shift in the pharmaceutical industry. As we move forward, continuous research and innovation will pave the way for a more sustainable and patient-focused pharmaceutical landscape.

## Data Availability

Data sharing is not applicable to this article.

## Abbreviations

ANN	Artificial neural network
AI	Artificial intelligence
API	Active pharmaceutical ingredient
ASP	Antisolvent precipitation
BWM	Ball wet milling
CESS^®^	Controlled expansion of supercritical solution
CMAs	Critical material attributes
CNS	Central nervous system
CPPs	Critical process parameters
CQAs	Critical quality attributes
3D	Three-dimensional
Dexi	Dexibuprofen
DMT	Disease-modifying treatment
DRZ	Dorzolamide hydrochloride
EAE	Experimental autoimmune encephalomyelitis
EUD	Eudragit
FD	Freeze-dried
FDA	Food and Drug Administration
GA	Glatiramer acetate
GI	Gastrointestinal
hERG	Human ether-a-go-go-related gene
HME	Hot-melt extrusion
HPH	High-pressure homogenization
HPMC	Hydroxypropyl methylcellulose
IOP	Intraocular pressure
ISGS	In situ gelling system
MD	Molecular dynamics
MIMO	Multiple-input–multiple-output
MISO	Multiple-input–single-output
MF	Microfluidic technology
ML	Machine learning
MRE	Mean relative error
MS	Multiple sclerosis
NanoPRX	Nanoformed piroxicam
NN	Neural network
NAP	Naproxen
NBCD	Non-biological complex drug
PEO-PPO-PEO	Poly(ethylene oxide)-Poly(propylene oxide)-Poly(ethylene oxide)
PAL	Palmatine HCL
PAT	Process analytical technology
PDI	Polydispersity index
PVP	Polyvinyl pyrrolidone
QbD	Quality by design
RTD	Room temperature-dried
SDS	Sodium dodecyl sulfate
SVM	Support vector machines
SSE	Semi-solid extrusion
TEER	Transepithelial electrical resistance
TIM	Timolol maleate
TNF-α	Tumor necroses factor-α
